# Direct Synthesis of Branched Carboxylic Acid Functionalized Poly(1-octene) by α-Diimine Palladium Catalysts

**DOI:** 10.3390/polym9040122

**Published:** 2017-03-27

**Authors:** Lihua Guo, Chen Zou, Shengyu Dai, Changle Chen

**Affiliations:** 1 School of Chemistry and Chemical Engineering, Qufu Normal University, Qufu 273165, China; guolihua-qfnu@139.com; 2 Key Laboratory of Soft Matter Chemistry, Chinese Academy of Sciences, Department of Polymer Science and Engineering, University of Science and Technology of China, Hefei 230026, China; chen1215@mail.ustc.edu.cn

**Keywords:** α-diimine, palladium, α-olefins (*co*)polymerization, acrylic acid, steric effect

## Abstract

In this work, we studied propylene polymerization using some α-diimine palladium catalysts with systematically varied ligand sterics. In propylene polymerization, the ligand steric effect exhibits significant variations on the catalytic activity, polymer molecular weight, and branching density. However, the regio control for the polymer microstructure is poor. Furthermore, copolymerization of 1-octene with the highly challenging and biorenewable comonomer acrylic acid was investigated. High copolymer molecular weights and high comonomer incorporation ratios could be achieved in this system. This study provides a novel access for the direct synthesis of branched carboxylic acid functionalized polyolefins.

## 1. Introduction

**Scheme 1:**
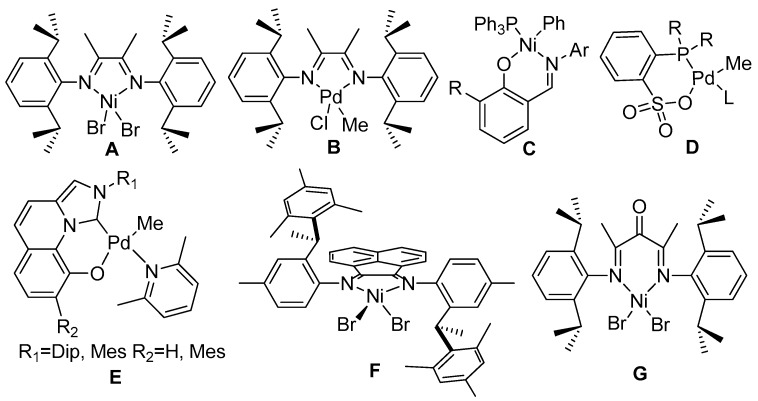
Representative late transition metal catalysts **A**–**G** that can catalyze olefin (*co*)polymerization.

Since the seminal work by Brookhart and coworkers in the 1990s [1,2], the α-diimine Ni(II) and Pd(II) catalysts (Scheme 1**A**,**B**) have achieved great successes in the area of olefin polymerization and copolymerization with polar monomers [3,4,5,6,7,8,9,10,11,12,13,14,15,16,17,18,19,20,21,22]. These catalysts possess many unique properties that are quite different from some other late transition metal catalysts such as salicylaldimine Ni(II) (Scheme 1**C**) [23,24,25,26,27,28] and phosphine-sulfonate Pd(II) (Scheme 1**D**) [29,30,31,32,33,34,35,36,37,38] catalysts. For example, various functional copolymers with acrylate, ester, and ether functional moieties can be prepared using α-diimine Pd(II) catalysts [39,40]. In addition, the strong chain walking ability of these α-diimine catalysts enables the synthesis of highly branched polymers. However, these catalysts also suffer from some limitations. For example, the resulting highly branched polymers and copolymers are usually amorphous and can only be applied in very specific areas. Furthermore, regio- and stereo-controlled α-olefins (e.g., propylene) polymerization and copolymerization with polar monomers are highly unlikely because of the fast chain walking process.

Stereo-regular polypropylene is an important engineering material produced on a large scale using early transition metal catalysts. However, the nonpolar nature of polypropylene leads to poor surface, adhesion, dye ability, and compatibility properties due to the absence of any polar functional group in its structure [41,42,43]. In order to improve these important properties, olefin/polar monomer copolymers are desirable in both industry and academia. Unfortunately, the high oxophilicity of early transition metals makes it very difficult to introduce polar groups into polypropylene. In contrast, late transition metal catalysts can tolerate polar groups and potentially produce functional polypropylene. Nozaki et al. reported the regio- and stereo-selective copolymerization of propylene with polar monomers using specially designed *N*-heterocyclic-carbene-based Pd(II) catalysts **E** or phosphine-sulfonate-based Pd(II) catalysts [35,44]. Generally, the formation of structural irregularities such as head-to-head/tail-to-tail defects and various branches in polypropylene is attributed to the poor regioselectivity of propylene insertion (1,2- or 2,1-) and very fast chain walking. In the majority of the cases, late transition metal catalysts (Scheme 1**F**,**G**) can only realize regio- and stereo-controlled propylene polymerization at very low temperatures (e.g., −60 °C) [45,46,47,48] in order to suppress the chain walking process. In the case of catalyst **G**, a chain end control mechanism is operative [48,49,50]. However, the polymerization activity and the polymer molecular weight would become very low at these temperatures. Therefore, the development of new catalysts that can exhibit good control under high temperatures is highly desired.

**Scheme 2:**
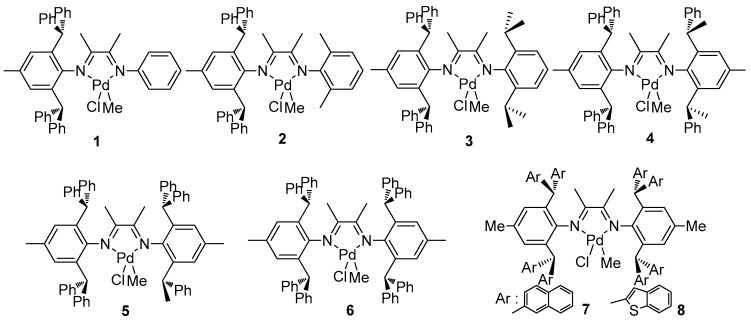
Dibenzhydryl-based α-diimine Pd(II) catalysts **1**–**8** bearing varied ligand sterics.

Our group recently reported the tuning of polyethylene and poly(1-hexene) microstructures using a series of Pd(II) catalysts **1**–**6** bearing both the dibenzhydryl moiety and systematically varied ligand sterics [51,52]. Furthermore, we synthesized high molecular weight polyethylene and functionalized polyethylene with branching densities as low as 6/1000C using sterically bulky α-diimine Pd(II) catalysts (Scheme 2, catalysts **7**,**8**) [53]. The unique microstructures of these (*co*)polymers were attributed to the slow chain walking feature of these Pd(II) catalysts. If the chain walking process could be sufficiently suppressed and 1,2- or 2,1-insertion of α-olefin could be selectively favored through ligand modifications, regio- and stereo-controlled α-olefin polymerization may be realized using these Pd(II) catalysts under certain reaction conditions.

In the above studies, very low polar monomer incorporation was observed because of the large difference in the insertion barriers between ethylene and the comonomers [51,52,53]. In this contribution, we decided to investigate the possibilities of regio-controlled polymerization of α-olefin using these α-diimine Pd(II) catalysts (**1**–**8**). In addition, the copolymerization of 1-octene with acrylic acid (AA) was also studied with the purpose of enhancing the polar monomer incorporation. Specifically, the influence of ligand steric effects on the catalytic properties and (*co*)polymer microstructures were investigated.

## 2. Experimental Section

### 2.1. General Information

All experiments were carried out under a dry nitrogen atmosphere using standard Schlenk techniques or in a glove-box. Deuterated solvents used for NMR were dried and distilled prior to use. ^1^H, ^13^C-NMR spectra were recorded by a Bruker Ascend Tm 400 spectrometer (Bruker, Karlsruhe, Germany) at ambient temperature unless otherwise stated. The chemical shifts of the ^1^H and ^13^C-NMR spectra were referenced to the residual solvent; coupling constants are in Hz. The molecular weight and the molecular weight distribution of the polymers were determined by gel permeation chromatography (GPC, Tosh, Tokyo, Japan) equipped with two linear Styragel columns at 40 °C using THF as a solvent and calibrated with polystyrene standards, and THF was employed as the eluent at a flow rate of 0.35 mL/min. Dichloromethane and toluene were purified by solvent purification systems. Catalysts **1**–**8** were prepared according to reported procedure [51,52,53]. All other reagents were purchased from commercial sources and used without purification.

### 2.2. Polymerizations of Propylene

Under an inert atmosphere, a 350 mL glass thick-walled pressure vessel was charged with sodium tetrakis(3,5-bis(trifluoromethyl)phenyl)borate (NaBAF), 23 mL toluene, and a magnetic stir bar. The pre-catalyst in 2 mL CH_2_Cl_2_ was injected, and the vessel was pressurized with propylene to initiate polymerization. After the desired amount of time, the polymerization was quenched via the addition of MeOH (5 mL), and the volatiles were removed. MeOH (10 mL) was added to precipitate the oily polymer and remove the MeOH-soluble impurities. The precipitate was dried in a vacuum oven to constant weight. Polymer branching density was determined by ^1^H-NMR. B = 1000 × 2(I_CH3_)/3(I_CH2+CH_ + I_CH3_). CH_3_ (m, 0.77–0.95 ppm); CH_2_ and CH (m, ca. 1.0–1.45 ppm).

### 2.3. Copolymerization of 1-Octene and AA

In a typical procedure, a round-bottom Schlenk flask with stirring bar was heated 1 h at 150 °C under vacuum and cooled to room temperature. A proper amount of NaBAF, freshly distilled 1-octene, and AA in CH_2_Cl_2_ was introduced into the flask. Polymerization was started by injecting the pre-catalyst solution (10 μmol, 2 mL CH_2_Cl_2_) into the reactor, and the total volume of the solution was kept at 10 mL. After the desired amount of time, the polymerization was terminated by adding 10 mL of the acidic ethanol (ethanol/HCl, volume ratio, 95:5). The solid polymer was filtered, washed with ethanol several times, and dried in vacuum at 40 °C to a constant weight. The oily polymer was washed with a large amount of methanol and redissolved in hot toluene. The polymer solution was filtered through alumina and silica to remove catalyst residues. After evaporation, the resulting polymers were dried under vacuum at 40 °C to a constant weight. The branches ending with functional groups were added to the total branches. AA% = 1/2 I_α-CH2_/[I_α-CH2_/2 + (I_CH3_ + I_CH2+CH_ + 1/2 I_α-CH2_)/16] × 100%. ^1^H-NMR (CDCl_3_): δ 2.35 (t, *J* = 7.5, 2H, α-CH_2_), 1.63 (quintet, *J* = 7.5, 2H, β-CH_2_), CH_2_ and CH (m, ca. 1.0–1.45 ppm). CH_3_ (m, 0.77–0.95 ppm); ^1^H-NMR (d^6^-benzene): δ 2.12 (t, *J* = 6.1, 2H, α-CH_2_), 1.64 (quintet, *J* = 6.1, 2H, β-CH_2_), CH_2_ and CH (m, ca. 1.0–1.45 ppm), CH_3_ (m, 0.77–0.95 ppm).

## 3. Results and Discussion

### 3.1. Propylene Polymerization Studies

Homopolymerization of propylene was investigated using catalysts **1**–**8** in combination with 1.2 equiv. of sodium tetrakis(3,5-bis(trifluoromethyl)phenyl)borate (NaBAF). The polymerization activity increased with ligand sterics from catalyst **1** to catalyst **3**. When the ligand sterics further increased from catalyst **4** to catalyst **8**, the activity decreased gradually. The polypropylene molecular weights had a trend similar to the ligand sterics. As a result, catalyst **3** showed the highest activity, with the turnover frequency of up to 1410 h^−1^ (Table 1, entry 3), and generated polypropylene with the highest molecular weight (*M*_n_ = 86.0 × 10^3^). The sterically very bulky substituents in catalysts **7** and **8** severely inhibited the coordination and insertion of propylene. The polymer dispersity index (PDI) of the polypropylenes were all larger than 1.4, and the theoretical number of polymer chains calculated from molecular weight and polymer yield ranged from 2 to 100, suggesting that the polymerization is not living under the current conditions.

This series of catalysts exhibited poor regio control for the polymerization of propylene. ^1^H-NMR spectroscopy analyses showed that the branching densities of the obtained polypropylenes were much lower than the theoretical value (333/1000C). The molecular weight of the product from catalyst **1** was too low (*M*_n_ = 0.7 × 10^3^) for accurate branching density calculation. The microstructure analysis of the polymers based on ^13^C-NMR showed the presence of only methyl, butyl, isobutyl, and long chain branches (see Supplementary Materials, Figure S4). These branches could be reasonably interpreted based on previous studies [54,55,56]. These polypropylene polymers are all amorphous and have no melting temperature (see Supplementary Materials, Figure S20). In general, both 1,2- and 2,1-insertion could occur in this system. 2,1-insertion followed by chain walking before the next insertion resulted in chain straightening by 3,1-enchainment, leading to reduced branching density [54,55,56]. In the case of catalysts **2** and **3**, the fraction of 3,1-insertion was lower than 30%, resulting in high branching densities (279/1000C and 235/1000C). For catalysts **4**–**6**, the 3,1-insertion was a major pathway, producing polymers with lower branching densities ranging from 153–174/1000C. Interestingly, the 3,1-insertion percentage and the polymer branching density for catalysts **7** and **8** were comparable with catalysts **4**–**6**. In this system, the competition between two insertion modes is mainly affected by the ligand steric effect.

### 3.2. 1-Octene-Acrylic Acid (AA) Copolymerization Studies

Very few late transition metal catalysts can catalyze olefin copolymerization with biorenewable comonomer acrylic acid, particularly because of the poisoning of the metal centers by the active proton in –COOH [57,58,59]. By using the sterically bulky catalysts **7** or **8**, no AA incorporation was observed in ethylene-AA copolymerization. Presumably, this is due to the large difference in the insertion barriers between ethylene and AA. Therefore, α-olefins were chosen to copolymerize with acrylic acid in this study because of their higher insertion barrier versus ethylene. Previously, copolymerization of α-olefins with methyl acrylate using α-diimine Pd(II) catalysts has been reported [40,60]. In propylene-AA copolymerization, very low incorporation ratio (ca. 0.1%) was found at 7 atm propylene and 1 mol/L AA. Subsequently, 1-octene and AA copolymerization was examined. It should be noted that the performance of these catalysts for 1-hexene and 1-octene polymerization has been studied by our group [51]. The data for 1-octene homopolymerization would be useful as a reference for this work (see Supplementary Materials, Table S1 and Figures S5–S9, S21–S24, S34–S37).

The copolymerization results are shown in Table 2. Catalyst **3** generates copolymers with the highest molecular weight (*M*_n_ = 40.4 × 10^3^). The catalytic activities of all catalysts were relatively low and had no clear trend. This is probably due to the low coordinating capability and high insertion barrier of 1-octene, as well as the poisoning effect of AA comonomer. Most interestingly, the percentage of acrylic acid incorporation was very high and could be tuned over a very wide range (1.07–15.7%) by the modification of ligand sterics. In this system, the rate of 1-octene insertion into a growing polymer chain was low compared to ethylene. Therefore, the high AA incorporation ratio is probably due to fact that the difference in the binding ability/insertion barrier is small between 1-octene and AA comonomer. Generally, the acrylic acid incorporation ratio decreased with ligand sterics from catalyst **1** to **6**. Catalysts **7** and **8** showed slightly higher incorporation ratio than catalyst **6**. At higher acrylic acid concentration (2 mol/L), the incorporation ratio could be increased significantly, almost twice as much as that at 1 mol/L (Table 2, entries 9 and 10). The branching densities of the copolymer products were 70–90/1000C and only varied slightly with the ligand sterics. The copolymer generated by catalysts **3**–**7** was semicrystalline, with a melting temperature approaching 72 °C. Mecking et al. reported that phosphinesulfonato palladium catalyst could copolymerize ethylene with AA to afford functionalized linear copolymers [56]. In this system, variously branched carboxylic acid functionalized polyolefins could be synthesized directly using α-diimine palladium catalysts. The PDIs of the polypropylenes were relatively large for catalyst **1** and **2** (Table 2, entries 1, 2, and 9), which may be ascribed to the very low polymer molecular weights (500 and 1100). The number of AA units in each polymer chain for these low molecular weight samples (Table 2, entries 1, 2, and 9) was ca. 0.7. For the high molecular weight samples, the number of AA units was in the range of 3.1–9.5 per polymer chain.

**Figure 1:**
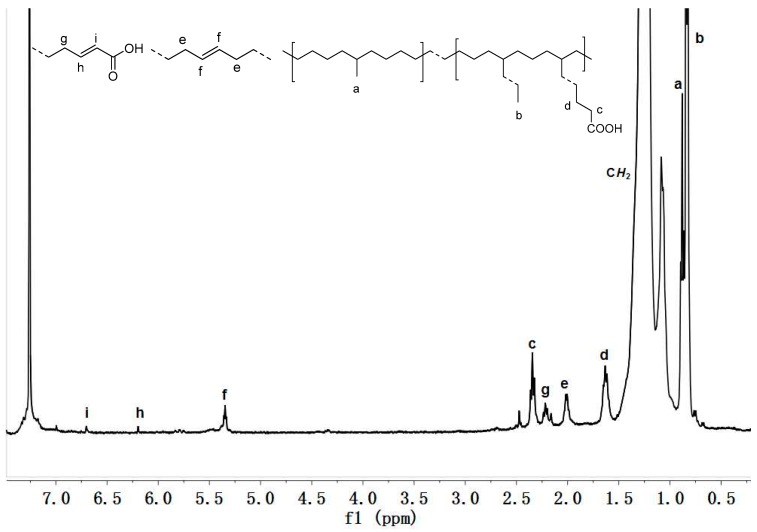
^1^H-NMR spectra of the copolymer obtained by Pd(II) catalyst (CDCl_3_, sample from Table 2, entry 10).

The ^1^H-NMR and ^13^C-NMR spectra of the copolymer products indicate the insertion of the AA comonomer (Figure 1, Figures S11–S13 in Supplementary Materials). The AA radical homopolymer is insoluble in CDCl_3_, while the copolymer is soluble. In CD_3_OD, the characteristic peak for radical AA homopolymer was at ca. δ 2.38, corresponding to CH_2_CH(COOH), and was very broad because of the irregularity of the repeating units (see Supplementary Materials, Figure S13). These results suggest the absence of AA radical homopolymerization. In the copolymer, the triplet at 2.35 ppm was assigned to the methylene hydrogen of the –CH_2_COOH moiety in the branches. In this system, the polar group was mainly located at the end of the branches instead of the main chain. According to the ^1^H-NMR spectrum analysis, both the –COOH groups in polymer branch and chain end are present. However, chain termination after AA insertion which results in the –COOH group in chain end is a minor pathway. For example, 18% of the chain end –COOH group was observed for catalyst **4**.

## 4. Conclusions

In conclusion, propylene polymerization and 1-octene/AA copolymerization using a series of α-diimine palladium catalysts were investigated. The aim was to systematically investigate ligand steric effects on the polymerization processes as well as the properties of the resulting polymer products. Unfortunately, the regio-controlled α-olefin polymerization could not be achieved in this system. However, the tuning in ligand sterics enables the tuning of the polymer microstructures such as branching density and molecular weight. The most interesting finding was that these sterically bulky α-diimine palladium catalysts could copolymerize 1-octene with biorenewable comonomer acrylic acid, with high comonomer incorporation and high copolymer molecular weights. However, it should be noted that the presence of high AA incorporation usually reduced the copolymer molecular weight.

## Figures and Tables

**Table 1:** Polymerization of propylene ^a^.

Entry	Cat.	Yield (g)	TOF ^b^ (h^−1^)	*M*_n_^c^ (×10^−3^)	PDI ^c^	*B* ^d^	3,1-Ins. ^e^ (mol %)
1	**1**	0.82	650	0.7	1.43	-	-
2	**2**	1.49	1180	56.6	1.67	279	16.3
3	**3**	1.78	1410	86.0	1.59	235	29.5
4	**4**	0.53	420	59.3	1.74	174	47.8
5	**5**	0.41	325	51.6	1.99	159	52.3
6	**6**	0.36	285	27.7	1.50	153	54.2
7	**7**	0.22	174	30.5	1.46	169	49.3
8	**8**	0.12	95	4.3	1.37	201	39.7

^a^ Conditions: 0.010 mmol pre-catalyst; propylene = 7 atm; 20 °C; 1.2 eq. sodium tetrakis(3,5-bis(trifluoromethyl)phenyl)borate (NaBAF); total volume of toluene, CH_2_Cl_2_: 25 mL; 3 h. ^b^ Turnover frequency = moles of substrate converted per mole of catalyst per hour. ^c^ Molecular weight (*M*_n_) and polymer dispersity index (PDI) was determined by gel permeation chromatography (GPC) in THF at 40 °C using polystyrene standards (see Supplementary Materials, Figures S30–S33). ^d^ *B* = branches per 1000 carbons, branching numbers were determined using ^1^H-NMR spectroscopy (see Supplementary Materials, Figures S1–S3). ^e^ %3,1-insertion calculated from the equation according to References [49,50]: %3,1-insertion = [(1000 − R)/(1000 + 2R)] × 100, R stands for the total methyl groups per 1000 methylene groups.

**Table 2:** 1-Octene (Oct)-acrylic acid (AA) copolymerization ^a^.

Entry	Cat.	[Oct] (M)	[AA] (M)	Yield (mg)	Act. ^b^	*X*_AA_^c^ (%)	*M*_n_^d^ (×10^−3^)	PDI ^d^	*B* ^e^	*T_m_* ^f^
1	**1**	2	1	201	6.7	15.7	0.5	3.78	93	-
2	**2**	2	1	141	4.7	3.59	2.3	2.34	85	-
3	**3**	2	1	253	8.4	2.77	40.4	2.03	98	14.1
4	**4**	2	1	231	7.7	1.95	35.7	1.37	66	35.7
5	**5**	2	1	165	5.5	1.28	28.2	1.41	58	56.0
6	**6**	2	1	203	6.8	1.07	34.8	1.52	60	72.0
7	**7**	2	1	48	1.6	1.82	26.3	1.30	79	25.2
8	**8**	2	1	102	3.4	1.26	8.6	1.60	84	-
9	**2**	2	2	50	1.7	7.24	1.1	3.23	95	-
10	**3**	2	2	156	5.2	5.89	8.5	1.59	80	-

^a^ Conditions: 0.010 mmol pre-catalyst; 1.2 eq. NaBAF; total volume of CH_2_Cl_2_ and AA: 10 mL; 3 h; 20 °C. ^b^ Activity (Act.) = 10^3^ g/(mol Pd·h). ^c^ *X*_AA_ = AA incorp. (mol %). ^d^ Molecular weight (*M*_n_) and polymer dispersity index (PDI) was determined by GPC in THF at 40 °C using polystyrene standards (see Supplementary Materials, Figures S38 and S39). ^e^ *B* = branches per 1000 carbons, branching numbers were determined using ^1^H-NMR spectroscopy (see Supplementary Materials, Figures S10–S19); the branches ending with functional groups are added to the total branches. ^f^ Melting temperature determined by differential scanning calorimetry (DSC) (see Supplementary Materials, Figures S25–S29).

## Supplementary Materials

The following are available online at www.mdpi.com/2073-4360/9/4/122/s1. The data of 1-octene polymerization (Table S1), NMR spectra of the polymers (Figures S1–S19), the typical GPC and DSC curves of polymer samples (Figures S20–S39).
