# Supplementary material for: Direct Synthesis of Branched Carboxylic Acid Functionalized Poly(1-octene) by α-Diimine Palladium Catalysts
Source: Polymers (Basel). 2017 Mar 27;9(4):122. doi: 10.3390/polym9040122 (PMC6432235; doi:10.3390/polym9040122)
Supplement: Supplementary file 1 [file polymers-09-00122-s001.pdf]

# Supplementary Materials: Direct Synthesis of Branched Carboxylic Acid Functionalized Poly(1-octene) by $\alpha$-Diimine Palladium Catalysts

**Lihua Guo, Chen Zou, Shengyu Dai and Changle Chen**

**Table S1.** Polymerization of 1-octene.[a]

| Entry | Cat. | Yield (g) | TOF[b] ($h^{-1}$) | $M_n$[c] ($\times 10^{-3}$) | PDI | $B$[d] | $T_m$[e] |
|---|---|---|---|---|---|---|---|
| 1 | 1 | 0.38 | 113 | 4.2 | 1.43 | 90 | oil[f] |
| 2 | 2 | 0.47 | 140 | 53.9 | 1.66 | 97 | 20.9 |
| 3 | 3 | 1.02 | 303 | 93.1 | 1.59 | 95 | 28.1 |
| 4 | 4 | 0.53 | 157 | 66.6 | 1.41 | 64 | 34.3 |
| 5 | 5 | 0.43 | 128 | 53.7 | 1.40 | 55 | 73.5 |
| 6 | 6 | 0.29 | 86 | 36.0 | 1.25 | 60 | 71.0 |
| 7 | 7 | 0.05 | 15 | 14.3 | 1.19 | 79 | oil[f] |
| 8 | 8 | 0.42 | 125 | 5.2 | 1.34 | 84 | oil[f] |

[a] Conditions: 0.010 mmol pre-catalyst, [1-octene] = 2 mol/L; 20 °C; 1.2 eq. NaBAF; total volume of $CH_2Cl_2$ and monomer: 10 mL; 3 h. [b] Turnover frequency = moles of substrate converted per mole of catalyst per hour. [c] Molecular weight was determined by GPC in THF at 40 °C using polystyrene standards. [d] B = branches per 1000 carbons, branching numbers were determined using $^1$H NMR spectroscopy. [e] Determined by differential scanning calorimetry (DSC). [f] Amorphous polymers (no $T_m$ from –50 to 120 °C).

The 1-octene polymerization was also investigated and the results are summarized in Table S1. With this longer $\alpha$-olefin monomer, more linear and less branched semicrystalline polymers were obtained through 2,1-insertion and 1,$\omega$-enchainment. The melting temperatures of the corresponding polymers increased with the decrease of branching density. The trend of activity, polymer molecular weight, and branching density with the ligand sterics was similar with propylene polymerization. Catalyst **3** showed the highest turnover frequency (303 $h^{-1}$, Table S1, entry 3) and generated poly(1-octene) with the highest molecular weight ($M_n$ = 93.1 × $10^3$). Different from the propylene polymerization, catalyst **5** generated polymer product with the lowest branching density (55/1000 C) and the highest melting point (73.5 °C). Most interestingly, the microstructure analysis of poly(1-octene) samples based on $^{13}$C-NMR spectroscopy showed the presence of only methyl and long chain branches (≥ hexyl) for all the catalysts (See supplementary materials, Figure S9). No ethyl, propyl, or adjacent methyl branches were detectable, indicating that the insertion occurred only into primary Pd-alkyl bonds.

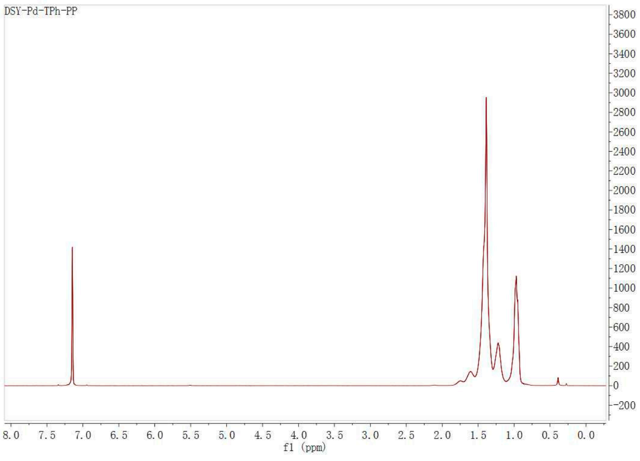


**Figure S1.** $^1$H NMR spectrum of the polymer from Table 1, entry 5 ($d^6$-benzene).

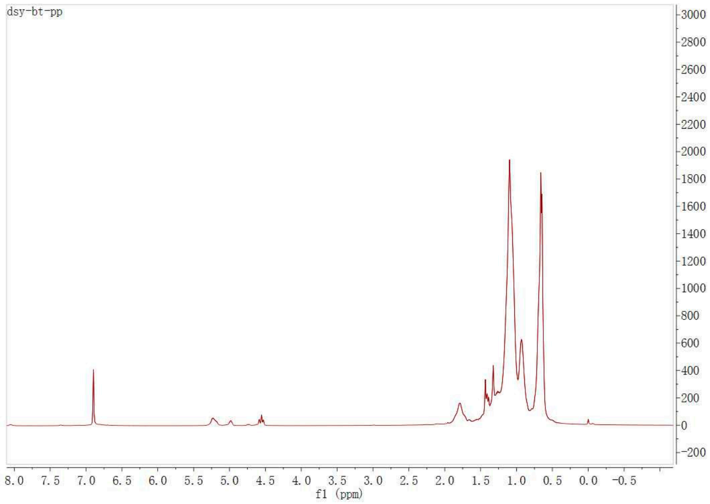


**Figure S2.** $^{1}$H NMR spectrum of the polymer from Table 1, entry 8 ($d^6$-benzene).

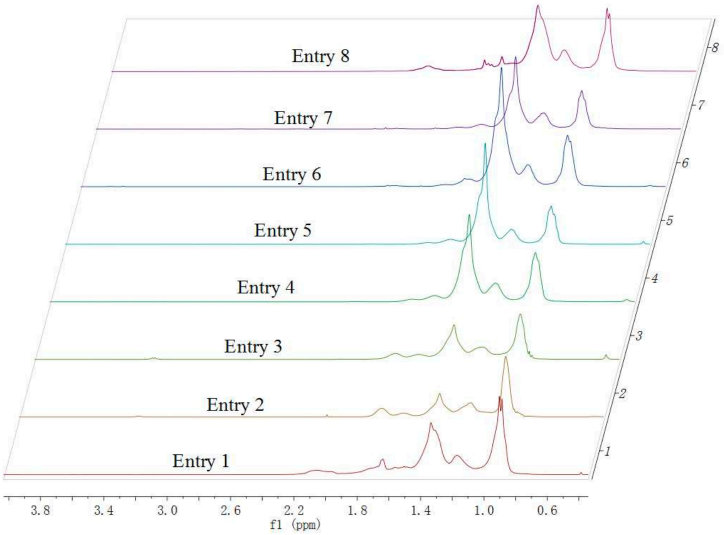


**Figure S3.** $^{1}$H NMR spectrum of the polymer from Table 1 ($d^6$-benzene).

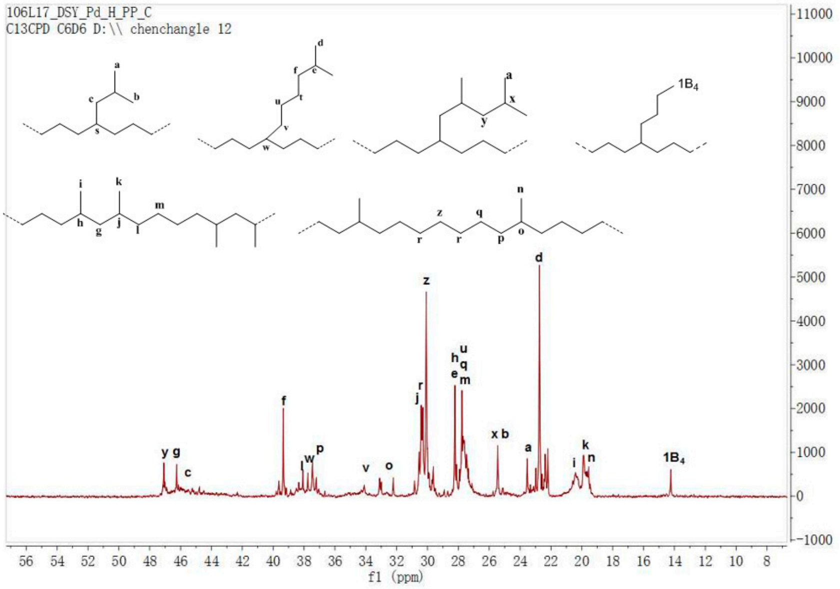


**Figure S4.** $^{13}$C NMR spectrum of the polymer from Table 1, entry 1 ($d^6$-benzene).

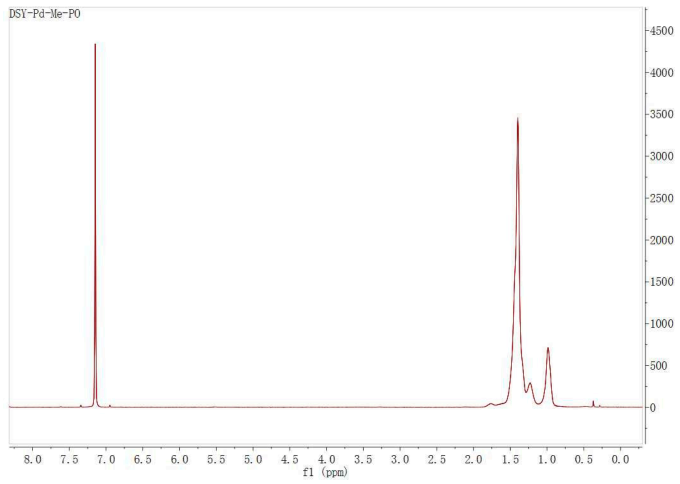


**Figure S5.** $^1$H NMR spectrum of the polymer from Table S1, entry 2 (d$^6$-benzene).

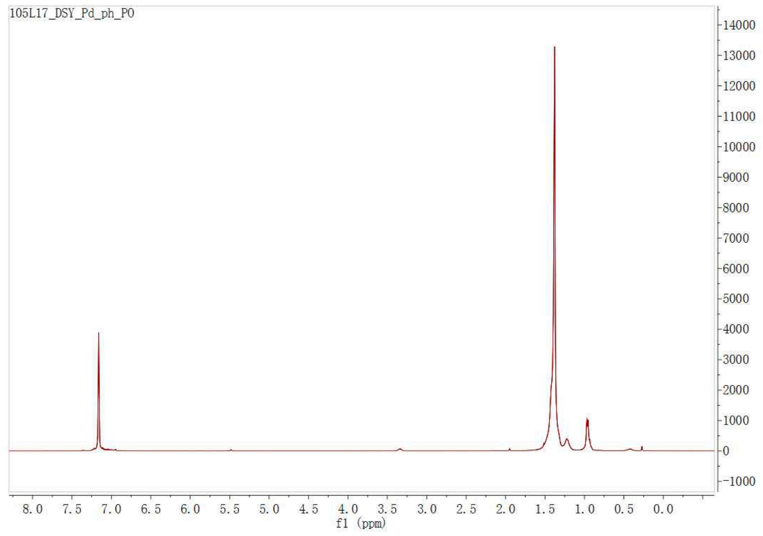


**Figure S6.** $^1$H NMR spectrum of the polymer from Table S1, entry 6 (d$^6$-benzene).

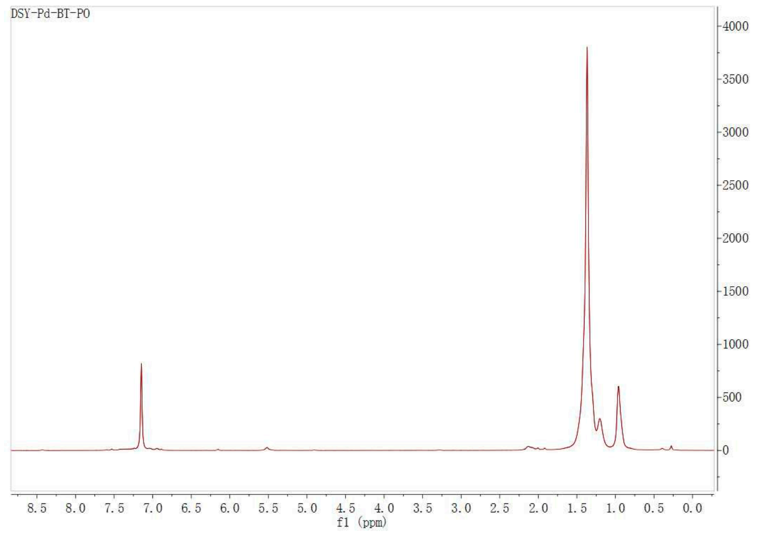


**Figure S7.** $^1$H NMR spectrum of the polymer from Table S1, entry 8 (d$^6$-benzene).

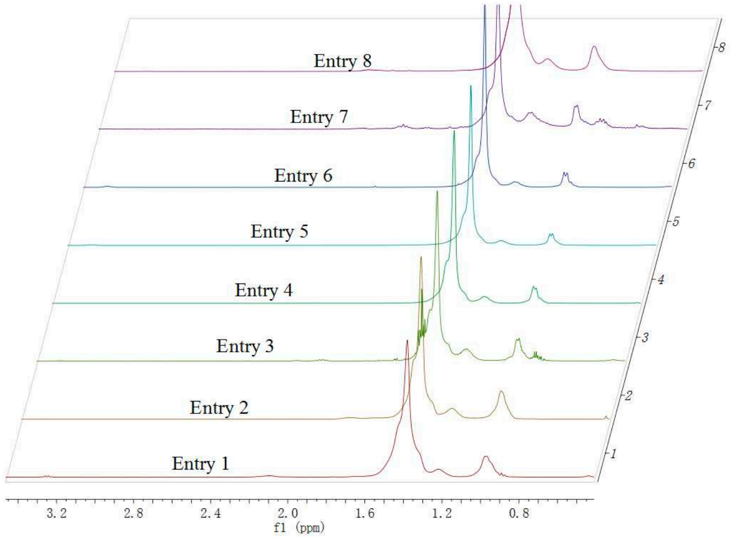


**Figure S8.** $^1$H NMR spectrum of the polymer from Table S1 (d$^6$-benzene).

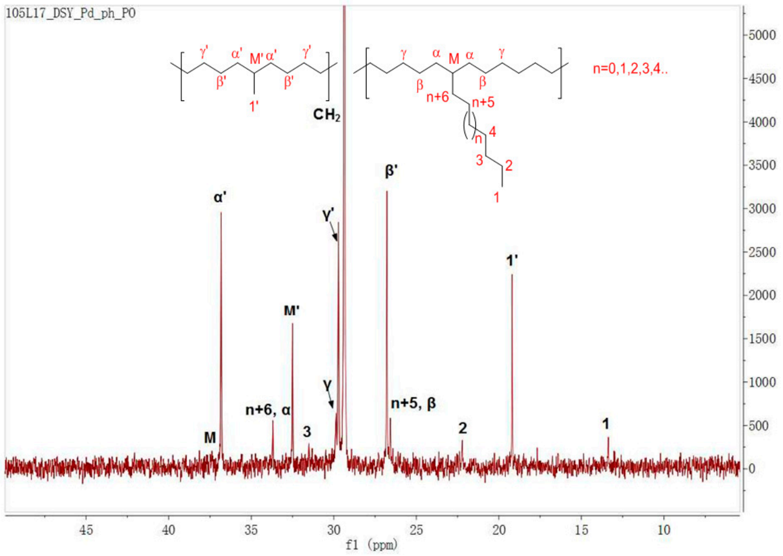


**Figure S9.** $^{13}$C NMR spectrum of the polymer from Table S1, entry 6 (d$^6$-benzene, 60$^o$C).

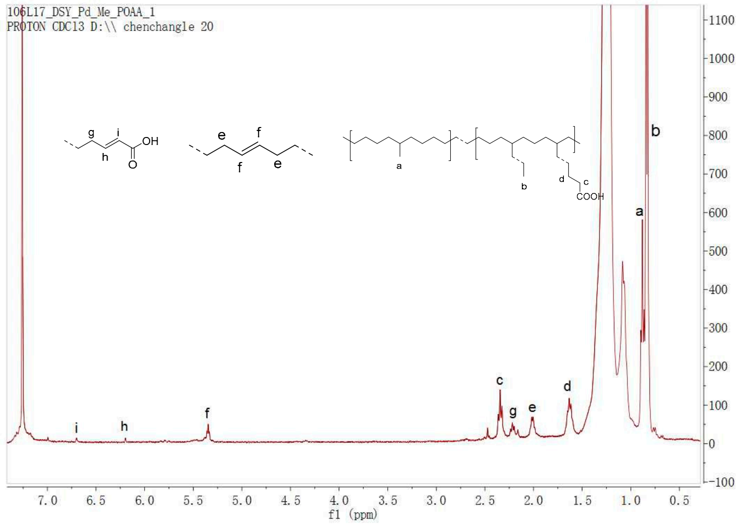


**Figure S10.** $^1$H NMR spectrum of the polymer from Table 2, entry 9 ($CDCl_3$).

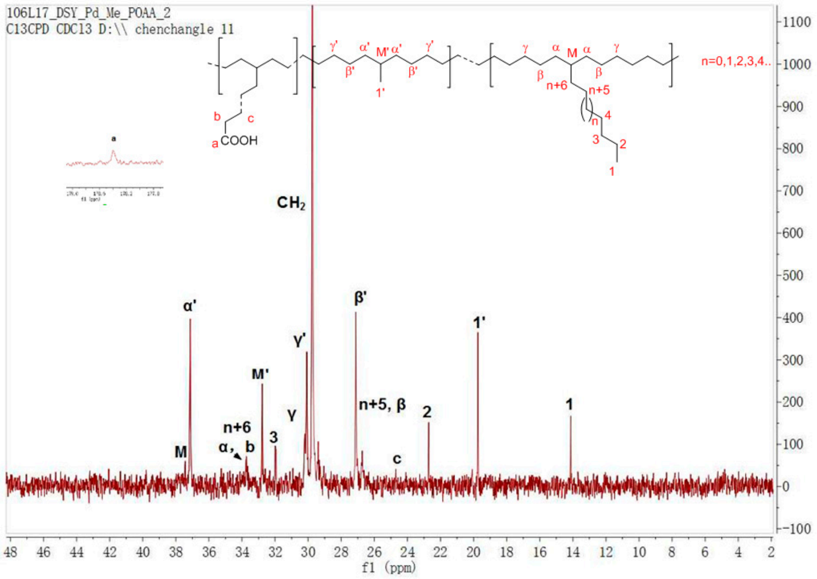


**Figure S11.** $^{13}C$ NMR spectrum of the polymer from Table 2, entry 9 ($CDCl_3$).

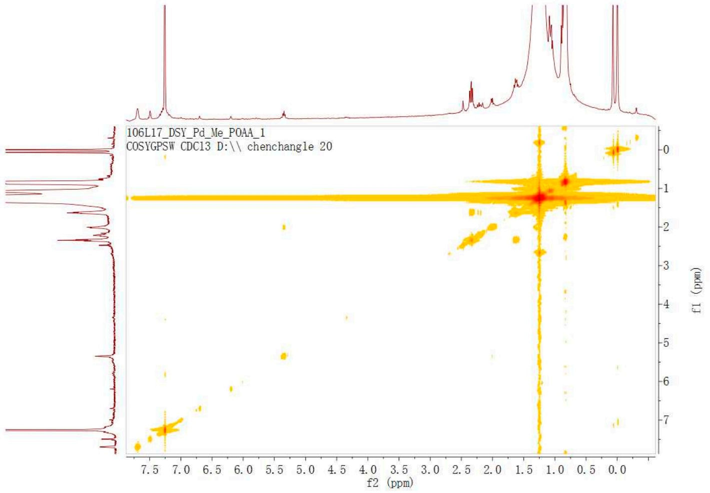


**Figure S12.** H-H COSY-NMR spectrum of the polymer from Table 2, entry 9 ($CDCl_3$).

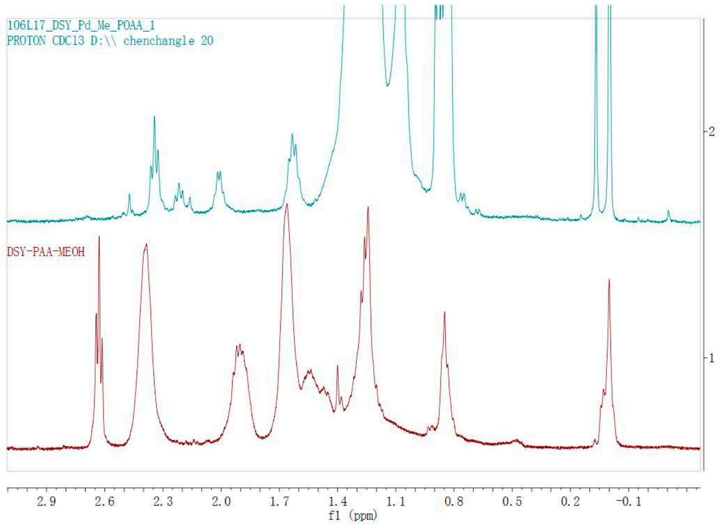


**Figure S13. Top:** $^{1}H$ NMR spectrum of the polymer from tTable 2, entry 9 ($CDCl_3$). **Bottom**: $^{1}H$ NMR spectrum of poly(acrylic acid) ($CD_3OD$).

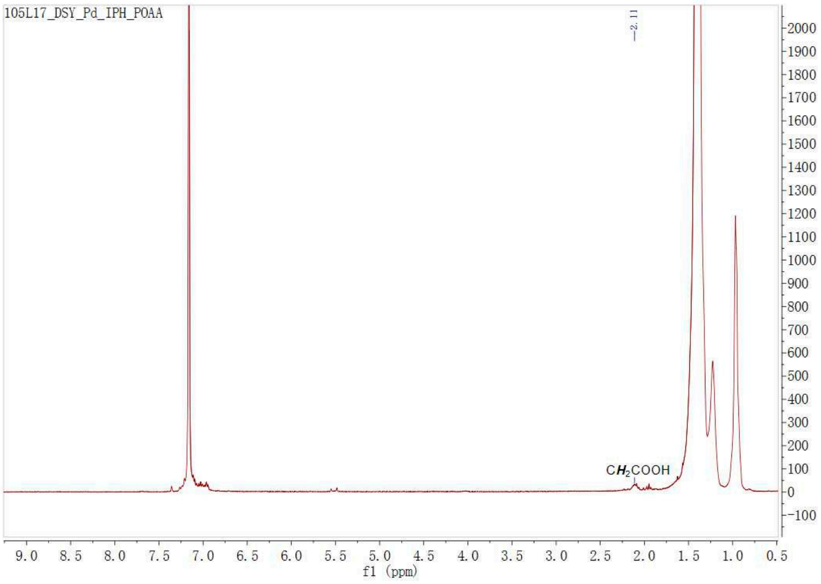


**Figure S14.** $^{1}$H NMR spectrum of the polymer from Table 2, entry 4 (d[6]-benzene, 60 °C).

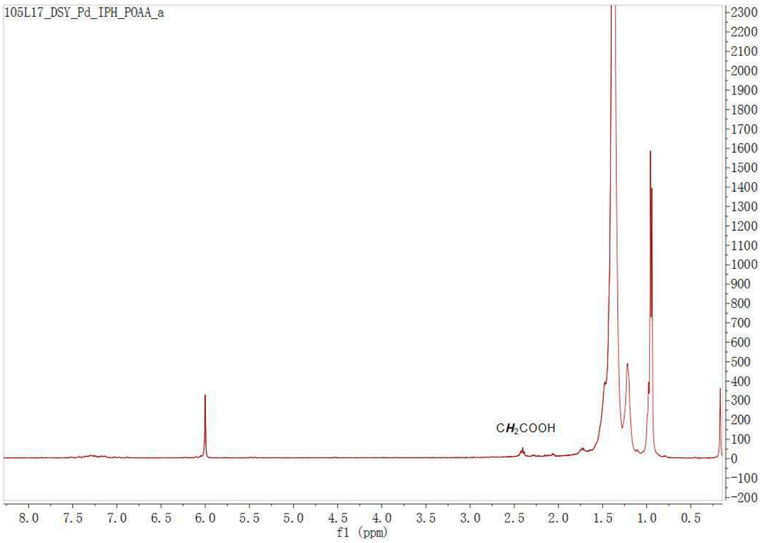


**Figure S15.** $^{1}$H NMR spectrum of the polymer from Table 2, entry 4 ($CDCl_2CDCl_2$, 120 °C).

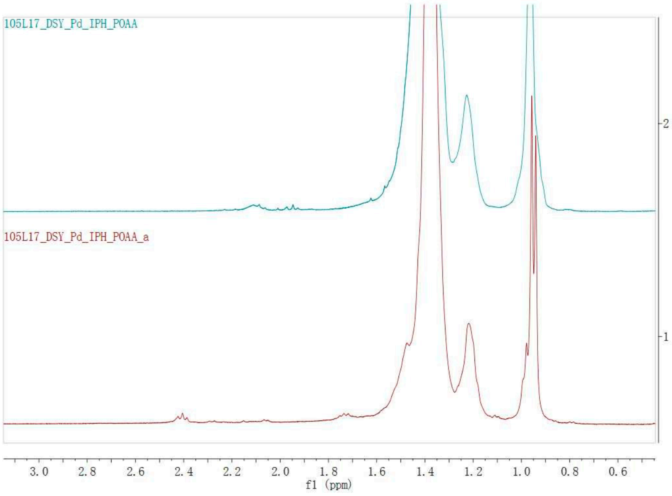


**Figure S16. Top:** $^{1}$H NMR spectrum of the polymer from Table 2, entry 4 (d[6]-benzene, 60 °C). **Bottom**: $^{1}$H NMR spectrum of the polymer from Table 2, entry 4 ($C_2D_2Cl_4$, 120 °C).

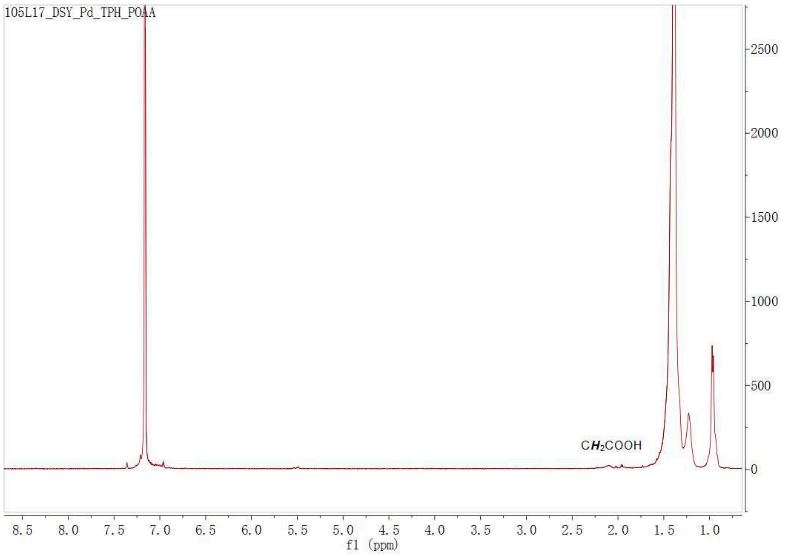


**Figure S17.** $^1$H NMR spectrum of the polymer from Table 2, entry 5 ($d^6$-benzene, 60 °C).

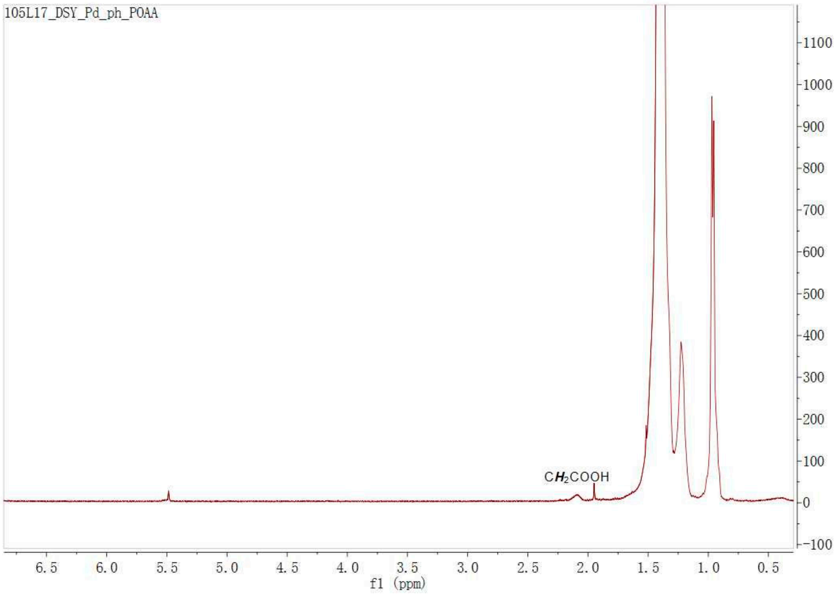


**Figure S18.** $^1$H NMR spectrum of the polymer from Table 2, entry 6 ($d^6$-benzene, 60 °C).

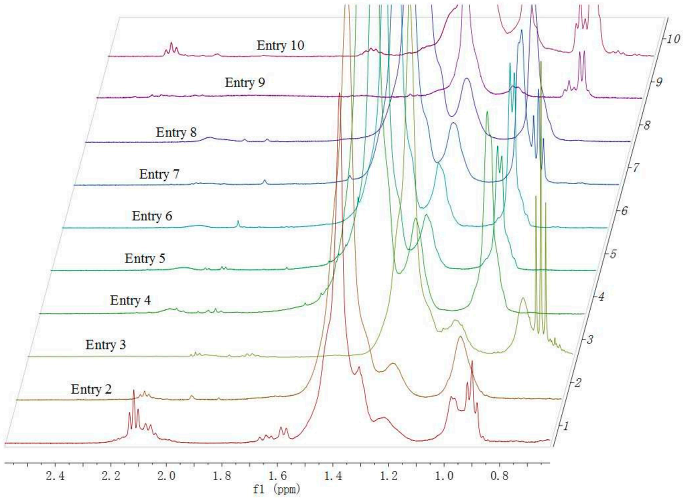


**Figure S19.** $^1$H NMR spectrum of the polymer from Table 2 (entry 1–8, $d^6$-benzene), (entry 9–10, $CDCl_3$).

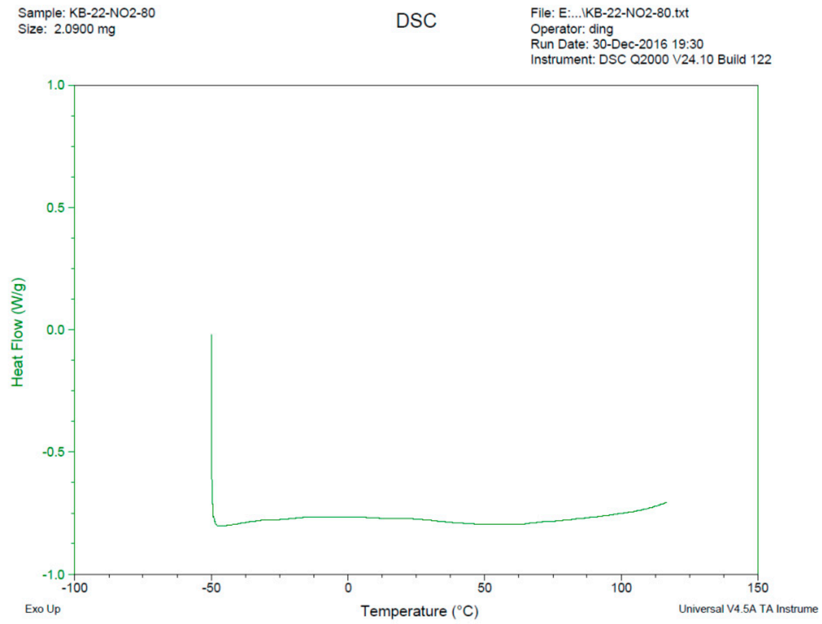


**Figure S20.** DSC of the polymer from Table 1, entry 3.

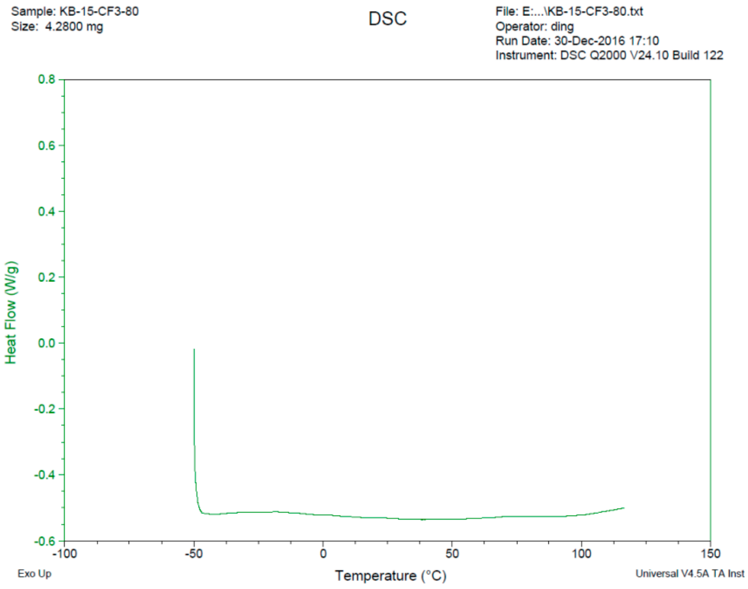


**Figure S21.** DSC of the polymer from Table S1, entry 1.

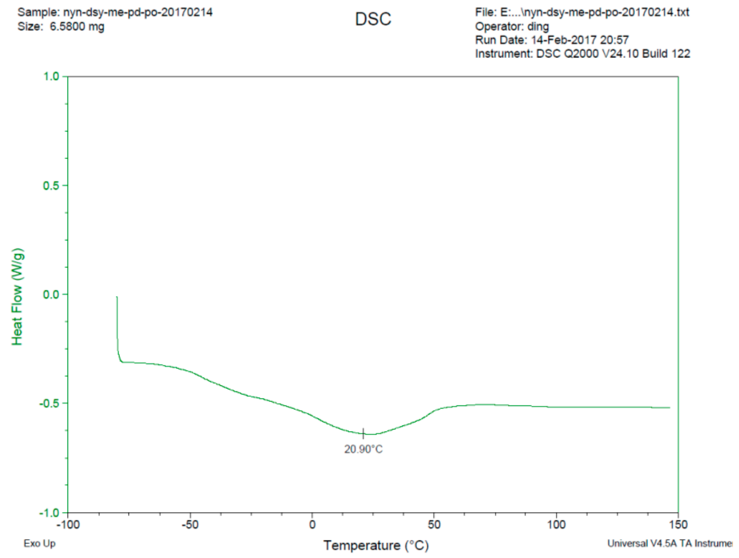


**Figure S22.** DSC of the polymer from Table S1, entry 2.

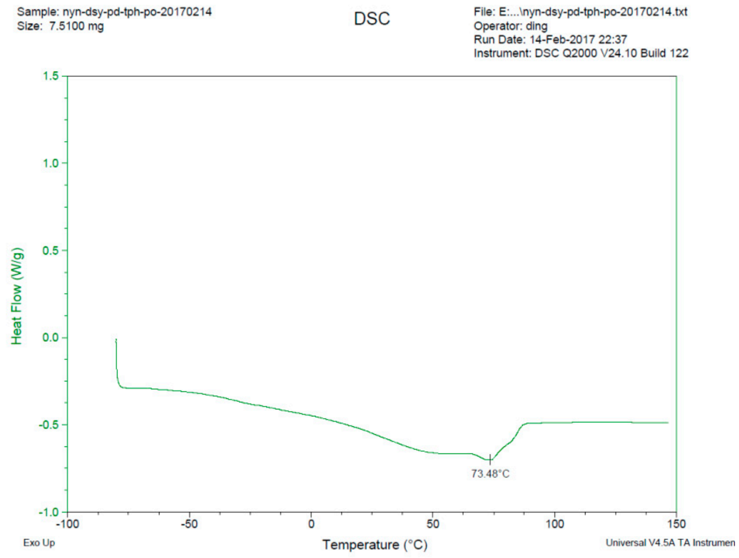


**Figure S23.** DSC of the polymer from Table S1, entry 5.

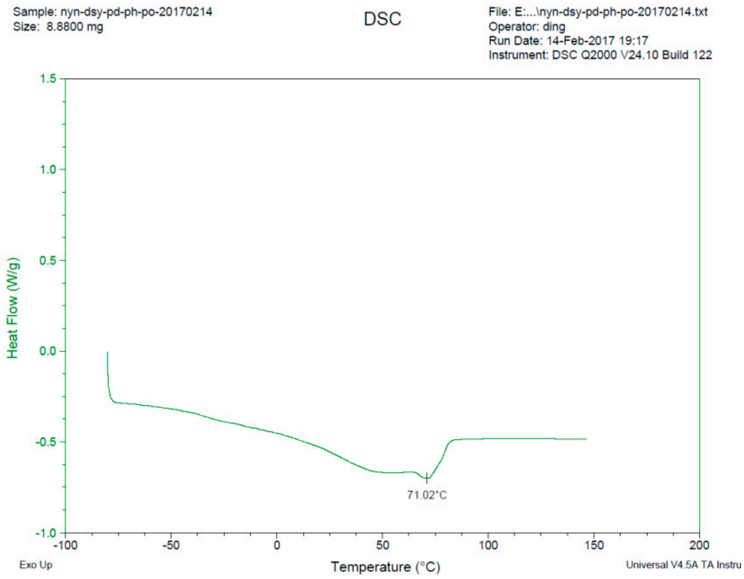


**Figure S24.** DSC of the polymer from Table S1, entry 6.

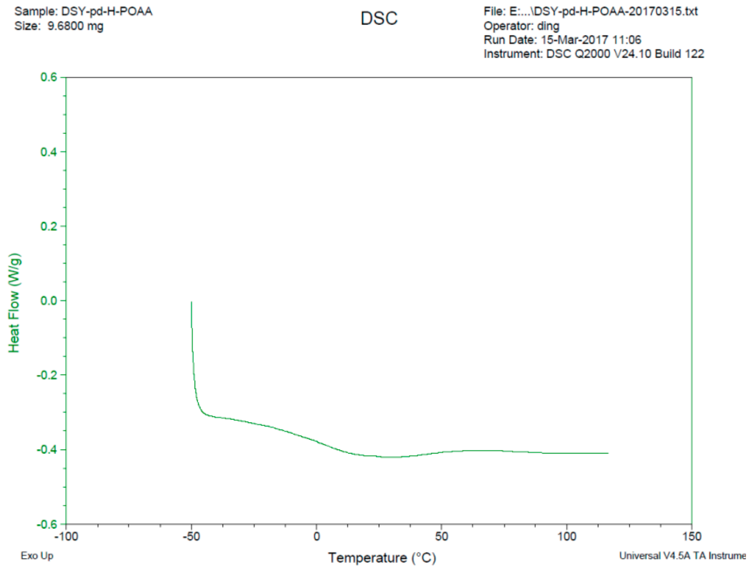


**Figure S25.** DSC of the polymer from Table 2, entry 1.

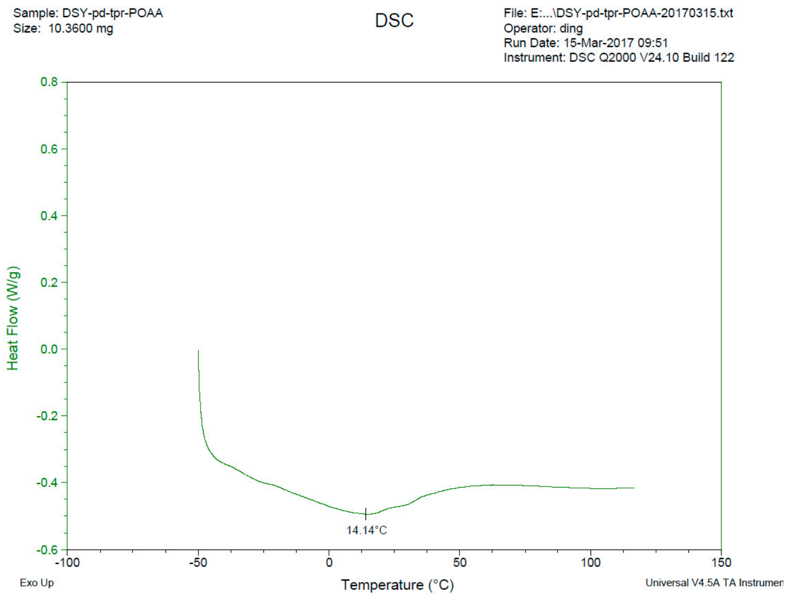


**Figure S26.** DSC of the polymer from Table 2, entry 3.

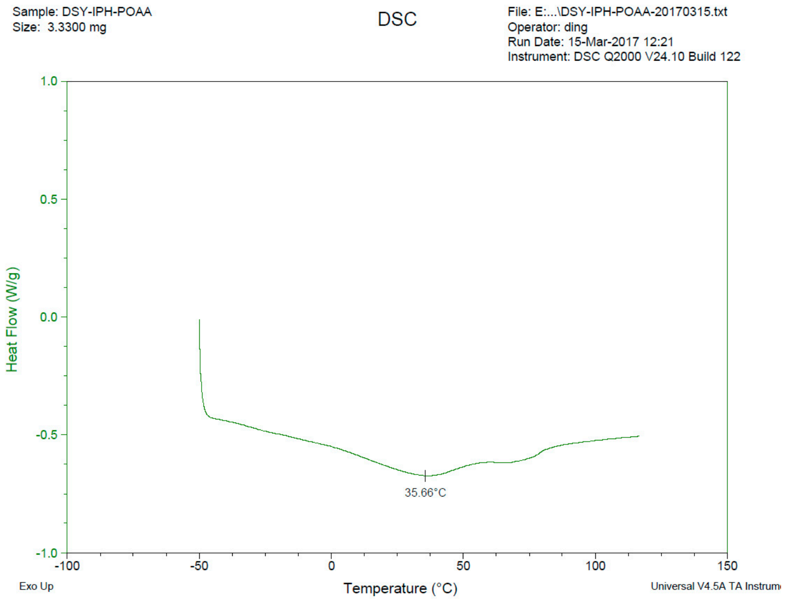


**Figure S27.** DSC of the polymer from Table 2, entry 4.

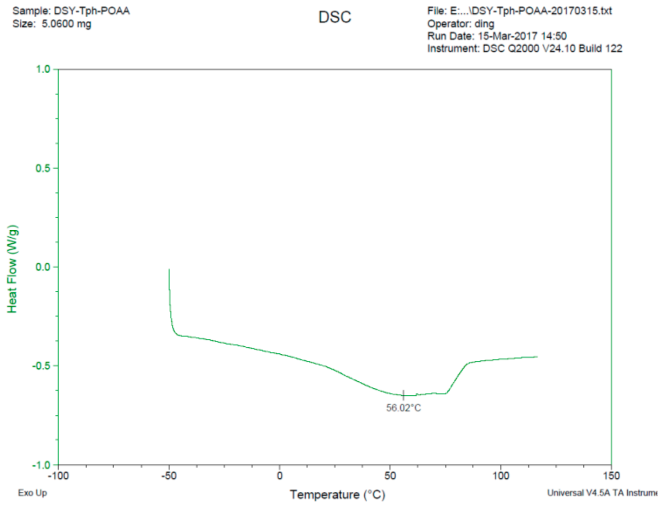


**Figure S28.** DSC of the polymer from Table 2, entry 5.

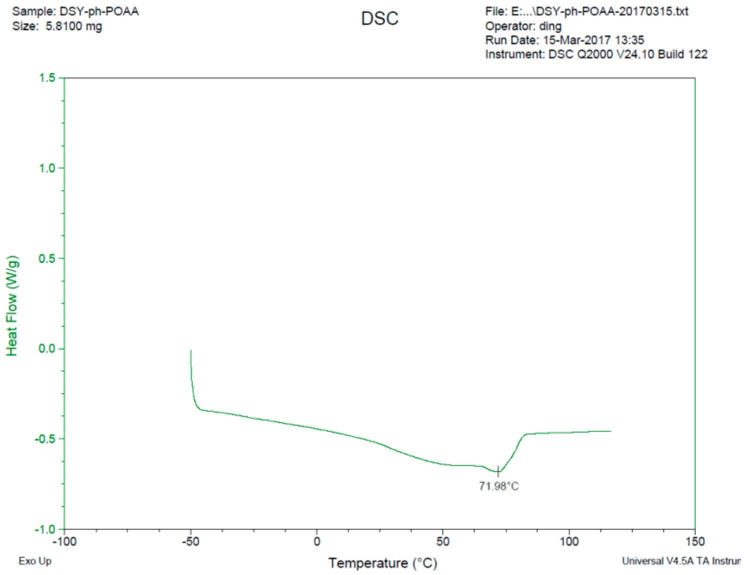


**Figure S29.** DSC of the polymer from Table 2, entry 6.

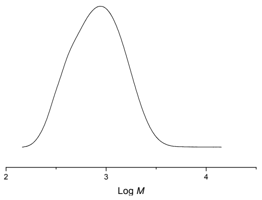


Result of molecular weight calculation (RI)

Peak 1 Base Peak

| | [min] | [mV] | [mol] | $M_n$ | 681 |
|---|---|---|---|---|---|
| Peak start | 8.610 | 0.971 | 14,232 | $M_w$ | 976 |
| Peak top | 10.508 | 164.293 | 870 | $M_z$ | 1,403 |
| Peak end | 11.728 | 1.209 | 144 | $M_{z+1}$ | 2,112 |
| | | | | $M_v$ | 976 |
| Height [mV] | | 163.177 | | $M_p$ | 871 |
| Area [mV·s] | | 10952.759 | | $M_z/M_w$ | 1.437 |
| Area% [%] | | 100.000 | | $M_w/M_n$ | 1.434 |
| [eta] | | 976.21958 | | $M_{z+1}/M_w$ | 2.163 |

**Figure S30.** GPC of the polymer from Table 1, entry 1.

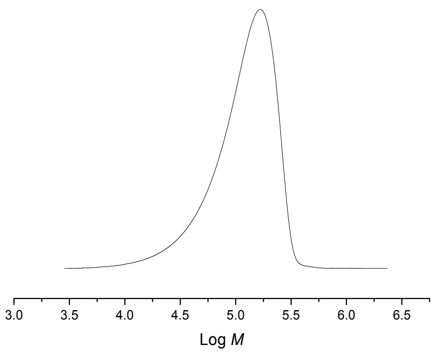


Result of molecular weight calculation (RI)
Peak 1 Base Peak

| | [min] | [mV] | [mol] | $M_n$ | 85,967 |
|---|---|---|---|---|---|
| Peak start | 5.147 | -0.038 | 2,327,316 | $M_w$ | 136,692 |
| Peak top | 6.940 | 239.341 | 166,206 | $M_z$ | 175,768 |
| Peak end | 9.703 | 1.057 | 2847 | $M_{z+1}$ | 220,024 |
| | | | | $M_v$ | 136,692 |
| Height [mV] | | 238.948 | | $M_p$ | 166,206 |
| Area [mV·s] | | 13,034.455 | | $M_z/M_w$ | 1.286 |
| Area% [%] | | 100.000 | | $M_w/M_n$ | 1.590 |
| [eta] | | 136,692.15043 | | $M_{z+1}/M_w$ | 1.610 |

**Figure S31.** GPC of the polymer from Table 1, entry 3.

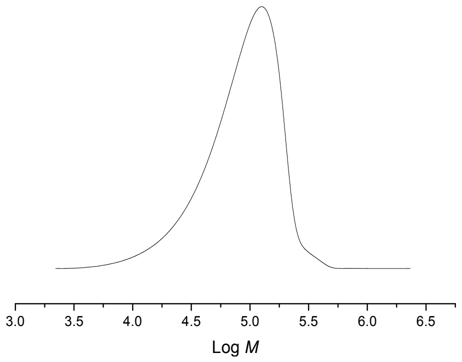


Result of molecular weight calculation (RI)
Peak 1 Base Peak

| | [min] | [mV] | [mol] | $M_n$ | 59,322 |
|---|---|---|---|---|---|
| Peak start | 5.640 | –0.036 | 1,126,008 | $M_w$ | 103,258 |
| Peak top | 7.123 | 203.261 | 126,902 | $M_z$ | 142,813 |
| Peak end | 9.878 | 1.635 | 2200 | $M_{z+1}$ | 183,348 |
| | | | | $M_v$ | 103,258 |
| Height [mV] | | 202.712 | | $M_p$ | 126,902 |
| Area [mV·s] | | 12,789.272 | | $M_z/M_w$ | 1.383 |
| Area% [%] | | 100.000 | | $M_w/M_n$ | 1.741 |
| [eta] | | 103,257.99615 | | $M_{z+1}/M_w$ | 1.776 |

**Figure S32.** GPC of the polymer from Table 1, entry 4.

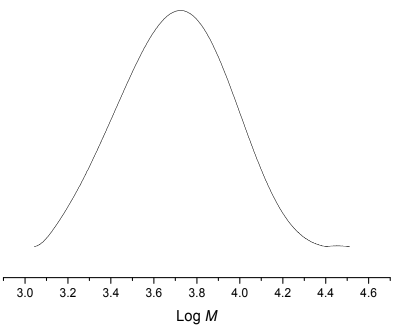


Result of molecular weight calculation (RI)
Peak 1 Base Peak

| | [min] | [mV] | [mol] | $M_n$ | 4304 |
|---|---|---|---|---|---|
| Peak start | 8.052 | 0.803 | 32,368 | $M_w$ | 5883 |
| Peak top | 9.278 | 169.785 | 5322 | $M_z$ | 7850 |
| Peak end | 10.347 | 41.304 | 1104 | $M_{z+1}$ | 9996 |
| | | | | $M_v$ | 5883 |
| Height [mV] | | 147.334 | | $M_p$ | 5322 |
| Area [mV·s] | | 9080.087 | | $M_z/M_w$ | 1.334 |
| Area% [%] | | 100.000 | | $M_w/M_n$ | 1.367 |
| [eta] | | 5883.28126 | | $M_{z+1}/M_w$ | 1.699 |

**Figure S33.** GPC of the polymer from Table 1, entry 8.

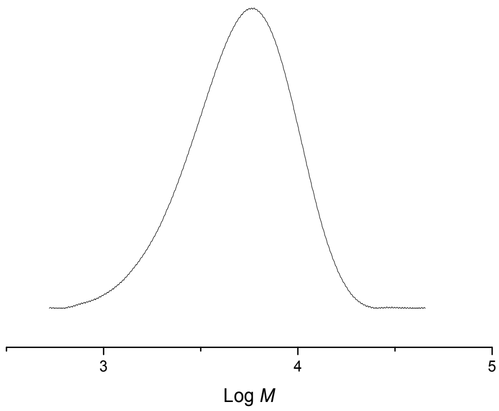


Result of molecular weight calculation (RI)
Peak 1 Base Peak

| | [min] | [mV] | [mol] | $M_n$ | 4180 |
|---|---|---|---|---|---|
| Peak start | 7.825 | 0.086 | 45,185 | $M_w$ | 5963 |
| Peak top | 9.202 | 22.526 | 5958 | $M_z$ | 7968 |
| Peak end | 10.853 | 2.030 | 524 | $M_{z+1}$ | 10,204 |
| | | | | $M_v$ | 5,963 |
| Height [mV] | | 21.556 | | $M_p$ | 5,958 |
| Area [mV·s] | | 1310.241 | | $M_z/M_w$ | 1.336 |
| Area% [%] | | 100.000 | | $M_w/M_n$ | 1.427 |
| [eta] | | 5963.05904 | | $M_{z+1}/M_w$ | 1.711 |

**Figure S34.** GPC of the polymer from Table S1, entry 1.

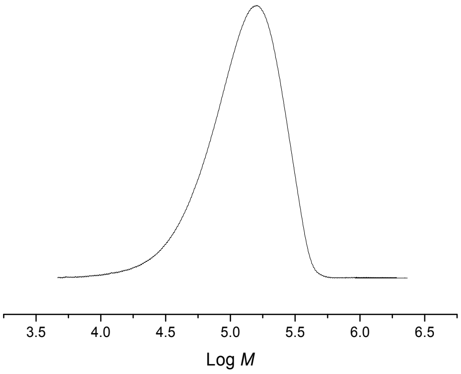


Result of molecular weight calculation (RI)
Peak 1 Base Peak

| | **[min]** | **[mV]** | **[mol]** | $M_n$ | **93,091** |
|---|---|---|---|---|---|
| Peak start | 5.277 | 0.142 | 1,922,055 | $M_w$ | 147,876 |
| Peak top | 6.973 | 22.523 | 158,249 | $M_z$ | 201,638 |
| Peak end | 9.373 | 0.181 | 4,627 | $M_{z+1}$ | 281,567 |
| | | | | $M_v$ | 147,876 |
| Height [mV] | | 22.365 | | $M_p$ | 158,249 |
| Area [mV·s] | | 1396.348 | | $M_z/M_w$ | 1.364 |
| Area% [%] | | 100.000 | | $M_w/M_n$ | 1.589 |
| [eta] | | 147,876.17847 | | $M_{z+1}/M_w$ | 1.904 |

**Figure S35.** GPC of the polymer from Table S1, entry 3.

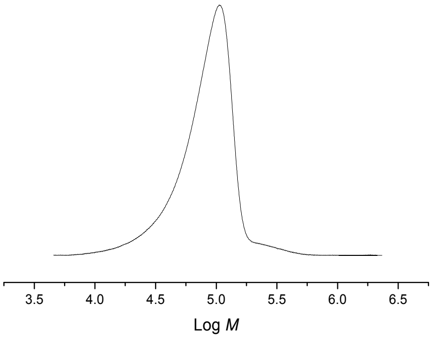


Result of molecular weight calculation (RI)
Peak 1 Base Peak

| | **[min]** | **[mV]** | **[mol]** | $M_n$ | **66,591** |
|---|---|---|---|---|---|
| Peak start | 5.208 | 0.025 | 2,125,402 | $M_w$ | 93,554 |
| Peak top | 7.247 | 29.457 | 105,837 | $M_z$ | 140,184 |
| Peak end | 9.383 | 0.234 | 4560 | $M_{z+1}$ | 380,096 |
| | | | | $M_v$ | 93,554 |
| Height [mV] | | 29.330 | | $M_p$ | 105,838 |
| Area [mV·s] | | 1186.394 | | $M_z/M_w$ | 1.498 |
| Area% [%] | | 100.000 | | $M_w/M_n$ | 1.405 |
| [eta] | | 93,554.14342 | | $M_{z+1}/M_w$ | 4.063 |

**Figure S36.** GPC of the polymer from Table S1, entry 4.

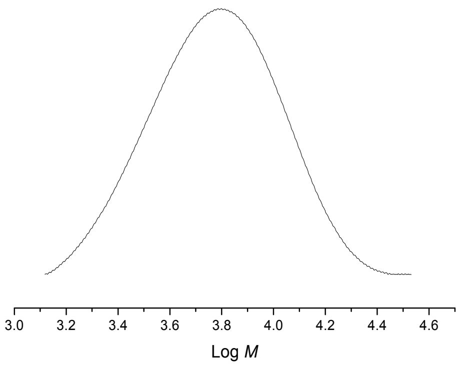


Result of molecular weight calculation (RI)
Peak 1 Base Peak

| | [min] | [mV] | [mol] | $M_n$ | 5,157 |
|---|---|---|---|---|---|
| Peak start | 8.018 | 0.259 | 33,996 | $M_w$ | 6912 |
| Peak top | 9.165 | 19.152 | 6288 | $M_z$ | 9038 |
| Peak end | 10.232 | 2.418 | 1308 | $M_{z+1}$ | 11,314 |
| | | | | $M_v$ | 6912 |
| Height [mV] | | 17.774 | | $M_p$ | 6288 |
| Area [mV·s] | | 1050.988 | | $M_z/M_w$ | 1.308 |
| Area% [%] | | 100.000 | | $M_w/M_n$ | 1.340 |
| [eta] | | 6912.11954 | | $M_{z+1}/M_w$ | 1.637 |

**Figure S37.** GPC of the polymer from Table S1, entry 8.

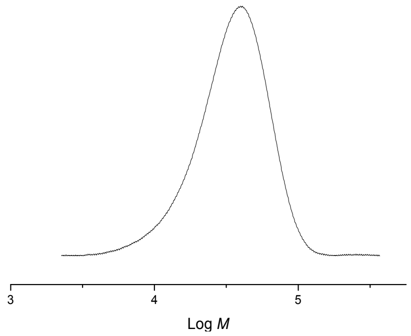


Result of molecular weight calculation (RI)
Peak 1 Base Peak

| | [min] | [mV] | [mol] | $M_n$ | 28,246 |
|---|---|---|---|---|---|
| Peak start | 6.397 | 0.540 | 369,758 | $M_w$ | 39,710 |
| Peak top | 7.912 | 16.877 | 39,774 | $M_z$ | 52,718 |
| Peak end | 9.863 | 0.998 | 2250 | $M_{z+1}$ | 73,785 |
| | | | | $M_v$ | 39,710 |
| Height [mV] | | 16.137 | | $M_p$ | 39,775 |
| Area [mV·s] | | 866.880 | | $M_z/M_w$ | 1.328 |
| Area% [%] | | 100.000 | | $M_w/M_n$ | 1.406 |
| [eta] | | 39,709.66949 | | $M_{z+1}/M_w$ | 1.858 |

**Figure S38.** GPC of the polymer from Table 2, entry 5.

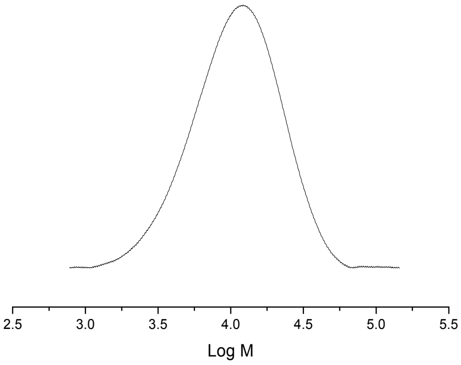

Result of molecular weight calculation (RI)
Peak 1 Base Peak

| | **[min]** | **[mV]** | **[mol]** | $M_n$ | **8,492** |
|---|---|---|---|---|---|
| Peak start | 7.032 | -0.062 | 145,230 | $M_w$ | 13,495 |
| Peak top | 8.715 | 17.607 | 12,194 | $M_z$ | 20,226 |
| Peak end | 10.588 | 1.122 | 774 | $M_{z+1}$ | 30,047 |
| | | | | $M_v$ | 13,495 |
| Height [mV] | | 17.109 | | $M_p$ | 12,194 |
| Area [mV·sec] | | 1192.169 | | $M_z/M_w$ | 1.499 |
| Area% [%] | | 100.000 | | $M_w/M_n$ | 1.589 |
| [eta] | | 13,494.91892 | | $M_{z+1}/M_w$ | 2.227 |

**Figure S39.** GPC of the polymer from Table 2, entry 8.
